# Landscape of Transcriptional Deregulations in the Preeclamptic Placenta

**DOI:** 10.1371/journal.pone.0065498

**Published:** 2013-06-13

**Authors:** Daniel Vaiman, Rosamaria Calicchio, Francisco Miralles

**Affiliations:** INSERM U1016-CNRS UMR8104, Université Paris Descartes, Institut Cochin, Paris, France; Otto-von-Guericke University Magdeburg, Germany

## Abstract

Preeclampsia is a pregnancy disease affecting 5 to 8% of pregnant women and a leading cause of both maternal and fetal mortality and morbidity. Because of a default in the process of implantation, the placenta of preeclamptic women undergoes insufficient vascularization. This results in placental ischemia, inflammation and subsequent release of placental debris and vasoactive factors in the maternal circulation causing a systemic endothelial activation. Several microarray studies have analyzed the transcriptome of the preeclamptic placentas to identify genes which could be involved in placental dysfunction. In this study, we compared the data from publicly available microarray analyses to obtain a consensus list of modified genes. This allowed to identify consistently modified genes in the preeclamptic placenta. Of these, 67 were up-regulated and 31 down-regulated. Assuming that changes in the transcription level of co-expressed genes may result from the coordinated action of a limited number of transcription factors, we looked for over-represented putative transcription factor binding sites in the promoters of these genes. Indeed, we found that the promoters of up-regulated genes are enriched in putative binding sites for NFkB, CREB, ANRT, REEB1, SP1, and AP-2. In the promoters of down-regulated genes, the most prevalent putative binding sites are those of MZF-1, NFYA, E2F1 and MEF2A. These transcriptions factors are known to regulate specific biological pathways such as cell responses to inflammation, hypoxia, DNA damage and proliferation. We discuss here the molecular mechanisms of action of these transcription factors and how they can be related to the placental dysfunction in the context of preeclampsia.

## Introduction

Preeclampsia (PE) is a pregnancy complication affecting approximately 5–8% of pregnant women and capable of causing both maternal and fetal morbidity and mortality. The disease develops after 20 weeks of gestational age and is characterized by elevated maternal blood pressure (140 mmHg/90 mmHg) and proteinuria (>300 mg/24h), endothelial cells (ECs) dysfunction and systemic inflammation [1]. In addition, PE can lead to eclampsia (when convulsions develop), and may be associated with the HELLP syndrome (Hemolysis, Elevated Liver enzymes and Low Platelet count). Both conditions may induce severe complications such as cerebral hemorrhage, lung edema or liver hemorrhage and rupture**.** PE symptoms appear after 20 weeks of gestational age, but sometimes much later by the end of pregnancy, and even, quite surprisingly, post-partum [2]. Those PEs who initiate early are generally more severe (blood pressure >160 mmHg/110 mmHg and proteinuria >300 mg/24h) and associated to a greater rate of intrauterine growth retardation and of iatrogenic prematurity.

Defective placentation is generally described as being at the root of the disease. Several studies have established that in the developing preeclamptic placenta, the normal process of trophoblast invasion and remodeling of the uterine maternal spiral arteries is impaired. This default in placental development in early pregnancy results in reduced placental perfusion, placental oxidative stress and inflammation, with subsequent release of placental factors and debris into the maternal circulation. These circulating factors are supposed to cause a widespread ECs activation leading to the multisystem dysfunction characteristic of the maternal syndrome of PE [3], [4]. Since the placenta plays a central role in the development of the disease, identifying the molecular mechanisms altered in the preeclamptic placenta comparatively to the non-pathologic placenta is fundamental to understand the initiation and evolution of this disease. In this context microarray-based genome-wide transcriptional profiling was used in several studies based on the comparison of the preeclamptic and non-pathologic placenta as reviewed by Louwen and collaborators [5]. In general, similar categories of differentially-expressed genes were reported including genes involved in: vascular regulation, inflammation, cell proliferation, apoptosis, differentiation, and cellular metabolism. However, in some cases the results appeared controversial in respect to some of the genes of interest. These differences may originate from the type of PE, the sampling of the placenta, the gestational age, ethnicity, mode of delivery, the microarray platforms and the filtering and statistical analysis. To overcome these differences we compared the lists of modified genes extracted from the publicly available datasets on microarray experiments concerning the preeclamptic placenta. The intersection of these gene-expression data sets, considering both up- and down-regulated genes, allowed obtaining a minimal list of genes which are consistently modified in PE. Then, we have used this consensus list to explore the transcriptional mechanisms involved in preeclampsia-specific placental dysfunction. This strategy has been used recently by Tapia and coworkers to identify with success transcription factors (TFs) involved in endometrial receptivity [6]. Transcriptional mechanisms control the expression of genes mainly through the action of TFs. These proteins bind to the DNA regulatory sequences of the genes at specific sites known as transcription factor binding sites (TFBS). Usually, the transcriptional activity of a gene requires the binding of several TFs, which act cooperatively to activate or repress transcription [7]. Therefore, we have used several bioinformatic tools allowing detecting over-representation of TFBS and of sets of TFBS in the promoters of genes. This way we identified a number of TFs which are likely involved in the regulation of the set of consistently modified genes in PE. These TFs may be instrumental in the transcriptomic modifications undergone by the preeclamptic placenta and their involvement in this disease can now be tested in the wet laboratory.

## Materials and Methods

### Data Sets used in this Study

We searched the public DataSets assembled from the Gene Expression Omnibus (GEO) repository, to identify expression microarray datasets that compared the expression of preeclamptic versus normal placentas. The keywords: preeclampsia, placenta, microarrays and gene-expression, were used for this search. To be included in our study the microarray experiments had to be done with placental biopsies collected at delivery and at relatively comparable gestational ages (30–39 weeks). This allowed to identify six datasets (**Table 1**). The GEO accession numbers of the studies are: GSE10588, GSE4707, GSE30186, GSE25906, GSE24129 and GSE14722, [8], [9], [10], [11], [12], [13]. The data from each study were analyzed with Geo2R to identify genes significantly modified (*P*-value ≤0.05 and Log2 Fold Change = ±0.2). This generated a list of modified genes (up- and down-regulated) for each study. Subsequently the lists of modified genes were confronted using the GENOMATIX list comparison tool (Genomatix Software GmbH, Munich, Germany) to identify those genes which were consistently modified (up- or down-regulated genes). Those showing similar modification in at least 4 studies were considered relevant and included in two final lists (consistently up-regulated and down-regulated genes).

**Table 1:** Preeclamptic placenta microarrays analyzed in this study.

Study	GEO accession	PE samples	Co samples	PE/Co Gest. Age (Weeks)	Delivery	Microarrays plataform
Sitras et al., 2009 [9]	GSE10588	17	26	34/39	CS	ABI HGSM Version 2
Nishizawa et al., 2007 [12]	GSE4707	13	8	32/32	CS	Agilent-012391 Whole Human Genome Oligo Microarray G4112A
Meng et al., 2011 [10]	GSE30186	6	6	36/39	CS	Illumina HumanHT-12 V4.0
Tsai et al., 2011 [11]	GSE25906	23	37	33/37	Labor	Illumina human-6 v2.0
Nishizawa et al., 2011 [13]	GSE24129	8	8	34/38	CS	Affymetrix Human Gene 1.0 ST Array
Win et al., 2009 [14]	GSE14722	12	11	32/31	CS/Labor	Affymetrix Human Genome U133 Plus 2.0

* Gestational age (weeks).

### Functional Clustering

The list of genes consistently up- and down-regulated within the microarray datasets was submitted to the GENOMATIX GeneRanker tool for functional annotation and pathway analysis. This allowed gaining information on the biological significance of these genes.

### Identification of Over-represented TFBS in the Proximal Promoter of the Genes Consistently Modified in the Preeclamptic Placenta

The sequences of the proximal promoter of the genes associated with the preeclamptic placenta were retrieved from the Data Base of Transcriptional Start Sites (DBTSS), [14]. For the purposes of this study the proximal promoter was defined as the region comprised within 1000 base pairs (bp) upstream and 200 bp downstream of the transcriptional start site (TSS). These sequences were used to search for potential TFBS using the following free softwares: CREMAG, a web tool that searches over-represented TFBS in a set of sequences using the TRANSFAC and JASPAR vertebrate position-weight matrices [15]. The analysis was performed with the default parameters. We used a 70% conservation threshold and a maximum number of 20 most conserved TFBSs in non-coding regions between 1000 bp upstream and 200 bp downstream of the TSS. TELIS (Transcription Element Listening System) is a Java server-side application which identifies transcription-factor binding motifs (TFBMs) that are over-represented among the promoters [16]. It consists of two parts: PromoterScan and PromoterStats. PromoterScan finds the number of occurrences of specific TFBMs in promoters and stores the results in MySQL database. PromoterStats uses z-statistics to find matrices which are over-represented (or under-represented) on the specific differentially expressed promoter set. The transcription factor affinity prediction (TRAP) method calculates the affinity of transcription factors for DNA sequences on the basis of a biophysical model [17]. This method has proven to be useful for several applications, including for determining which transcription factors have the highest affinity in a set of sequences [18]. TFM-explorer is a program for analyzing regulatory regions of eukaryotic genomes. It takes a set of co-regulated gene sequences, and search for locally over-represented TFBS [19]. The algorithm proceeds in two steps: (i) it scans sequences for detecting all potential transcription factor binding sites, using weight matrices from JASPAR or TRANSFAC. (ii) it extracts significant clusters (region of the input sequences associated with a factor) by calculating a score function. The web tool TOUCAN uses the MotifScanner algorithm to search for potential TFBS in a set of sequences using the TRANSFAC or JASPAR vertebrate databases. The information obtained from the MotifScanner is subsequently processed by the statistics function of TOUCAN to identify over-represented TFBS [20], [21]. We used several different TFBS prediction software’s because these bioinformatics tools usually generate a number of false positives. Thus, only TFBS predicted by more than one tool were considered as true positives.

### Identification of Regulatory Modules

To identify common regulatory modules in a set of promoter sequences we used the Genomatix FrameWorker software. FrameWorker identifies significant complex models of TFBS present in the promoter sequences of a set of co-regulated genes. The models/FrameWorkers are defined as all the TFBs that occur in the same order and in a certain distance range in all (or a subset) of the input sequences. To determine the *P*-value of the models, a background promoter sequence set of 5000 human promoters is scanned with the models generated by the software. This allows calculating the probability to found the same models in a set of randomly selected promoters.

### Transcription Factors Interaction

TFs interactions were identified through the Search Tool for the Retrieval of Interacting Genes/Proteins (STRING) database v9.0. This database contains known and predicted physical and functional protein-protein interactions [22]. STRING was used in the protein mode, and only interactions based in experimental protein-protein interaction and curated databases with confidence levels over 0.5 were considered.

## Results

### Identification of Genes Consistently Associated with the Preeclamptic Placenta

The intersection of the lists of modified genes extracted from the microarray studies of the preeclamptic placenta yielded a short list of genes being consistently modified in the different studies. We identified a total 98 modified genes of which 67 were up-regulated and 31 down-regulated. **Table 2** (up-regulated) and **Table 3** (down-regulated) show a selection of consistently modified genes in PE (Complete lists are provided as **Tables S1** and **S2**). The most consistently up-regulated genes were LEP and FLT1 (present in the totality of studies), followed by QPCT, SIGLEC6, ENG, BCL6, INHA, EBI3, PAPP2 and HTRA1 (found modified in five studies). The most consistently down-regulated gene modified in all the studies was CLDN1. Followed by genes present at least in four out of six studies including among others ABAT, SOD1, GCLM, APLN, ABCG2, and NR2F1.

**Table 2:** Partial list of consistently up-regulated genes in the preeclamptic placenta.

Gene	EntrezGene ID	GSE4707	GSE24129	GSE10588	GSE25906	GSE14722	GSE30186
LEP	3952	1	1	1	1	1	1
FLT1	2321	1	1	1	1	1	1
HTRA1	5654	1	1	1	1	1	-
QPCT	25797	1	1	1	1	1	1
SPAG4	6676	1	1	1	1	1	-
INHA	3623	1	1	1	1	1	-
PAPPA2	60676	1	1	1	1	1	-
SIGLEC6	946	1	1	1	1	1	-
ENG	2022	1	1	1	1	1	-
INHBA	3624	1	1	1	1	1	-
BCL6	604	-	1	1	1	1	-
SLC26A6	65010	-	1	1	1	-	1
GREM2	64388	1	1	1	1	1	-
EBI3	10148	1	1	1	1	1	-
HTRA4	203100	1	1	1	1	-	-
FSTL3	10272	1	1	1	1	1	-
BHLHE40	8553	1	1	1	1	1	-

The table shows some of the consistently up-regulated genes in the six preeclamptic placenta microarray studies analyzed. The microarrays are identified by their GEO data set accession number (GSE). (1) Indicates modified in the microarrays, (-) Indicates not-modified. *Complete consensus list of up-regulated genes in the preeclamptic placenta is provided as Table S1.

**Table 3:** Partial list of consistently down-regulated genes in the preeclamptic placenta.

Gene	EntrezGene ID	GSE4707	GSE24129	GSE10588	GSE25906	GSE14722	GSE30186
CLDN1	9076	1	1	1	1	1	1
ABAT	18	1	1	1	-	1	1
MFF	56947	-	1	1	1	1	-
GCLM	2730	-	1	1	1	1	1
F13A1	2162	-	1	1	1	1	-
SOD1	6647	-	1	1	1	1	-
APLN	8862	1	1	-	1	-	1
ABCG2	9429	-	1	1	1	1	-
GOT1	2805	-	1	1	1	1	-
SLC23A2	9962	-	1	1	1	-	1
OLFML3	56944	-	1	-	1	1	1
LEPREL1	55214	1	1	1	-	1	-
BHLHE41	79365	-	1	1	-	1	-
FAM101B	359845	1	1	1	1	-	-
NR2F1	7025	-	1	1	1	1	-

The table shows some of the consistently down-regulated genes in the six preeclamptic placenta microarray studies analyzed. The microarrays are identified by their GEO data set accession number (GSE). (1) Indicates modified in the microarrays, (-) Indicates not-modified. *Complete consensus list of down-regulated genes in the preeclamptic placenta is provided as Table S2.

### Functional Clustering Analysis

We then used the GENOMATIX Gene Ranker software to perform functional and network analysis of the consistently modified genes. This made it possible to identify functional gene classifiers (**Table 4** **and** **5**) and pathways (**Table 6**) that are significantly enriched in the preeclamptic placenta. Among the up-regulated genes the most significant functional categories were signaling and signal transduction, the regulation of biological quality, interferon-gamma biosynthetic process, the regulation of B cell differentiation and cell proliferation. The list of down-regulated genes was enriched in transcripts involved in the response to regulation of sulfur metabolism, blood vessel size and blood circulation, cellular homeostasis, and the responses to chemical stimulus and oxidative stress. The pathways with the highest scores include the peroxisome proliferative activated receptor alpha, lipid, hypoxia inducible factor 1, FMS like receptor tyrosine kinase 3 and vascular endothelial growth factor pathways. In addition, we noticed that in at least three out of the six microarray studies some of the consistently modified genes in the preeclamptic placenta encode TFs. Among the up-regulated genes we found: LIMD1 (LIM domain-containing protein 1), BHLHE40 (Basic helix-loop-helix family member e40), VDR (Vitamin D 1,25-dihydroxyvitamin D3 receptor), CEBPA (CCAAT/enhancer binding protein, alpha), BCL6 (B-cell CLL/lymphoma 6), ARID3A (AT rich interactive domain 3A) and NRIP1 (Nuclear receptor interacting protein 1). Among the down-regulated genes: TFDP2 (Transcription factor Dp-2), ZFAND5 (Zinc finger, AN1-type domain 5), BHLHE41 (Basic helix-loop-helix family, member e41), and NR2F1 (Nuclear receptor subfamily 2, group F, member 1). These TFs were also included in further analyses.

**Table 4:** Biological processes annotation clusters for up-regulated genes as reported by the GENOMATIX webtool.

Database	Functional annotation	N° of genes	Genes	*P*-Value
GO:0023052	Signaling	36	HTRA1, HEXB, CEBPA, DDR1, LYN, CYP26A1, ENG, APLP2, EBI3, KIT, HLPDA, SYDE1, RASEF, PREX1, BAD, VDR, INSIG1, FLT1, LHB, FSTL3, SCARB1, LIMD1, MIF, BHLHE40, SIGLEC6, TREM1, LEP, GREM2, ERRFI1, NRIP1, INHBA, CORO2A, ERO1L, INHA, SH3BP5, NEK111	1.73E-06
GO:0065008	Regulation of biological quality	25	HTRA1, HEXB, DDR1, LYN, CYP26A1, ENG, APLP2, BCL6, KIT, GAPDH, EZR, BAD, VDR, KIF2A, LHB, HTRA4, SCARB1, BHLHE40, PAPPA2, TREM1, LEP, INHBA, INHA, HK2, PROCR	3.42E-06
GO:0030099	Myeloid cell differentiation	7	CEBPA, LYN, KIT, FSTL3, LEP, INHBA, INHA7	7.47E-06
GO:0007165	signal transduction	32	tHTRA1, CEBPA, DDR1, LYN, CYP26A1, ENG, APLP2, EBI3, KIT, SYDE1, RASEF, PREX1, BAD, VDR, INSIG1, FLT1, LHB, FSTL3, SCARB1, LIMD1, MIF, TREM1, LEP, GREM2, ERRFI1, NRIP1, INHBA, CORO2A, ERO1L, INHA, SH3BP5, NEK111	1.72E-05
GO:0045577	B cell differentiation	3	BAD, INHBA, INHA1	1.81E-05
GO:0022414	Reproductive process	15	GPX3, HEXB, DDR1, APLP2, KIT, IGSF8, VDR, FLT1, LHB, LEP, NRIP1, INHBA, SPAG4, INHA, HK2	2.74E-05
GO:0045072	Interferon-gamma biosynthesis	3	EBI3, INHBA, INHA2	2.98E-05
GO:0008283	Cell proliferation	16	CEBPA, DDR1, LYN, ENG, EBI3, KIT, HLPDA, IGSF8, BAD, VDR, INSIG1, FLT1, SCARB1, MIF, INHBA, INHA	5.99E-05

**Table 5:** Biological processes annotation clusters for down-regulated genes as reported by the GENOMATIX webtool.

Database	Functional annotation	N° of genes	Genes	*P*-Value
GO:0006790	Sulfur compound metabolism	4	GCLM, SOD1, ENPP1, GOT1	1.41E-04
GO:0050880	Regulation of blood vessel size	3	GCLM, SOD1, APLN	5.71E-04
GO:0006536	Glutamate metabolic process	2	GCLM, GOT1	4.03E-04
GO:0006979	Response to oxidative stress	4	GCLM, SOD1, SLC23A2, SEPP1	4.85E-04
GO:0008015	Blood circulation	4	GCLM, ABAT, SOD1, APLN	1.34E-03
GO:0042311	Vasodilation	2	APLN, SOD1	2.17E-03
GO:0065008	Regulation of biological quality	10	HSD17B1, GCLM, SOD1, APLN, ABCG2, F13A1, ABAT, NRCAM, ENPP1, GOT1	3.15E-03

**Table 6:** Signal transduction pathways as reported by the GENOMATIX web tool.

Pathway	N° of genes	Observed genes	*P*-value
Peroxisome Proliferative Activated Receptor Alpha	5	VDR, LHB, SCARB1, LEP, NRIP1	5.04E-05
Lipid	8	LYN, PREX1, EZR, SCARB1, LEP, ARID3A, HK2, PROCR	2.97E-04
Hypoxia inducible Factor 1, alpha subunit	4	NDRG1, FLT1, MIF, ERO1L	2.02E-03
FSM Like Receptor Tyrosine Kinase 3	3	CEBPA, LYN, KIT	2.71E-03
Vascular Endothelial Growth Factor	5	ENG, KIT, PREX1, FLT1, ERO1L	3.08E-03
Nuclear Receptor Subfamily 1, Group H	2	VDR, SCARB1	4.60E-03
BCL2 Associated Athanogene	2	BAD, VDR	8.15E-03
Chemokine (CXC Motif) Receptor 4	3	KIT, PREX1, MIF	9.09E-03
TEK Tyrosine Kinase	2	ENG, FLT1	9.83E-03
Nuclear Receptor Subfamily 2, GroupF	1	NR2F1	4.56E-03

### Identification of Over-represented TFBS among the Consistently Modified Genes

Co-expressed groups of genes are expected to share regulatory elements which are responsible of the co-regulation. Thus, to identify the putative common regulatory elements the lists of up- and down-regulated genes were analyzed with bioinformatics tools. First the proximal promoter sequences of the genes (1000 bp up-stream and 200 bp downstream of the TSS) were retrieved from the DBTSS data base, and subsequently analyzed with several public TFBS detection tools: CREMAG, TELIS, TRAP, TFM-Explorer, and TOUCAN. Only those TFBS showing a *P* value ≤0.05 for their observed frequency versus their predicted frequency were considered. The results of these analysis are listed in **Table 7** and **Table 8**. The most significant over-represented TFBS found in the up-regulated genes list correspond to NFkB (Nuclear factor kappa B), RREB1 (Ras responsive element binding protein 1), SP1 (Specificity protein 1), ARNT (Aryl hydrocarbon receptor nuclear translocator), CREB1 (cAMP responsive element binding protein 1) and AP-2 (Activating enhancer-binding protein 2). In the down-regulated list the most significant over-represented TFBS are MZF1 (Myeloid zinc finger 1), E2F1 (E2F transcription factor 1), MEF2A (Myocyte enhancer factor 2A) and NFYA (Nuclear transcription factor Y, alpha). Some TFBS, such as SP1, E2F1, ARNT, and MZF1 appear over-represented in both up- and down-regulated genes.

**Table 7:** Transcription factor binding sites over-represented in the consensus list of up-regulated genes.

TFBS detection tools						
CREMAG (1)	CREMAG (2)	TELIS (2)	TRAP (1)	TFM-explorer (1)	TFM-explorer (2)	TOUCAN (2)
**CREB1**	**REST**	**GC**	INSM1	**KLF4**	**SP1**	**SP1**
CEBPA	OLF1	**AP2**	**AP2**	**SP1**	**GC**	**AP2**
NFIL3	CEBPA	**MZF1**	**KLF4**	**ARNT**	**MZF1**	**PAX5**
HLF	**ATF2**	**NFKB**	EBF1	**MYC**	**AP2**	**E2F1**
ELK1	**CREB1**	**ARNT**	**PAX5**	**RREB1**	**NFKB**	**NFKB**
**PAX5**	**E2F1**	CREL	**RREB1**	**EGR1**	**RREB1**	MAZR
PAX4	E4BP4	IK2	**TP53**	MAX	**TP53**	NFYA
	**ATF**	**SP1**	Tcfcp2l1	MYC	**MYCMAX**	**ATF2**
	**MZF1**		ESR1	HIF1A	USF	NRF2
	SRY		**EGR1**	**SPZ1**	HOX13	NGFIC
	STAT3		**NFKB**		CAP	**E2F1**
	OCT1B		**MYCMAX**		BARBIE	**SPZ1**
	**SP1**		**MZF1**		**ARNT**	AP4
	**AP1**		**REST**			**EGR1**
			MYF			**RREB1**
			**AP1**			**GC**
			ESRRB			**CREB1**
			PLAG1			**STAF**
			**ARNT**			
			HNF4A			
			ZFX			
			**E2F1**			
			**CREB1**			
			**SPZ1**			
			ESR2			

TFBS with a prevalence *P* value ≤0.05 are shown. (1) and (2) indicate that the TFBS weight matrices used for the analysis were respectively JASPAR or TRANSFAC. TFBS predicted by more than one analysis tool appear in bold.

**Table 8:** Transcription binding sites over-represented in the consensus list of down-regulated genes.

TFBS detection tools						
CREMAG (1)	CREMAG (2)	TELIS(2)	TRAP (1)	TFM-explorer (1)	TFM-explorer (2)	TOUCAN
**NFYA**	AP1	**COUP**	**MZF1**	**NFYA**	CEBP	TAXCREB
**MZF1**	SRF	**MEF2A**	**E2F1**	KLF4	**AP4**	**NFYA**
PAX4	NRSF	**MZF1**	CTCF	HLTF	**NFYA**	**ARNT**
**E2F1**	GC	IRF2	NFATC2	PAX6	**FOXF1**	**COUP**
**Evi1**	**OCT-1B**	**AP4**	MYF	FOXL1	OCT-6B	**OCT-1B**
	**SP1**	**SP1**	Zfp423	MAFB	Gfi-1	CREBP1
	**MZF1**	AP2	**EGR1**	**TBP**	ISRE	**E2F1**
	**ARNT**	NKX25	**FOXD1**	**Evi1**	**MEF2A**	**MEF2A**
	**E2F1**	**E2F1**	**SP1**	**FOXD1**	TATA	HNF4
			SPI1	**MEF2A**	**OCT-1B**	SREBP1
			**MEF2A**	FOXA2	GATA-1	PAX5
			NR3C1	**EGR1**	**ARNT**	XFD2
			PDX1		HNF3B	**AP4**
			NFE2L2		En-1	**FOXF1**
			MYB			
			**NFYA**			
			**TBP**			

TFBS with a prevalence *P* value ≤0.05 are shown. (1) and (2) indicate that the TFBS weight matrices used for the analysis were respectively JASPAR or TRANSFAC. TFBS predicted by more than one analysis tool appear in bold.

### Search for Regulatory Modules in TFs Consistently Modified in Preeclampsia

**Figure 1:**
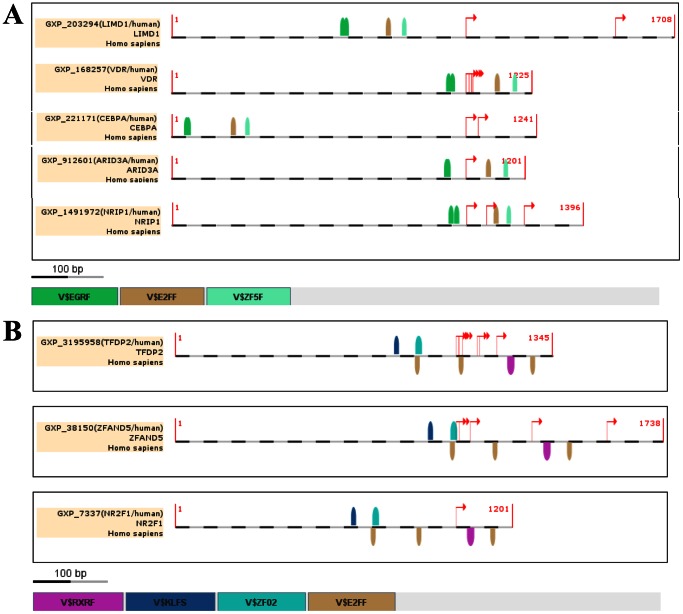
Transcription factors modules in the promoter sequences of TFs consistently modified in PE. The TFs consistently modified (either up- or down-regulated ) in PE were analyzed with the Genomatix FrameWorker software to identify common regulatory modules. (**A**) We identified seven common significant four-element modules in the promoters of five out of seven consistently up-regulated TFs. Here we show the most significant (*P*≤7.8×10^−11^) regulatory module composed of TFBs for EGRF, E2FF and ZF5F. (**B**) We identified one significant 6-element regulatory module (*P≤*1.9×10^−10^) in the promoters of tree out of four TFs down-regulated in the preeclamptic placenta. This module is composed of TFBS for E2FF, RXRF, KLFs and ZF02.

The intersection of the microarrays of preeclamptic placentas indicates that a few TFs appear consistently modified at the transcriptional level (either up- or down-regulated). Thus, these transcriptionally co-regulated TFs could share common regulatory elements in their promoters. These elements are often organized into defined motifs (frameworks) of two or more TFBs which are located in the promoter of the genes in a specific orientation, separated by a given distance and working in concert. We used the Genomatix FrameWorker software to identify putative regulatory modules among the TFs consistently modified in the preeclamptic placenta. Among the promoter sequences of the TFs consistently up-regulated we got seven significant models (i.e. modules) of three elements present in the promoter of five genes out of seven. The most significant model (*P*≤7.82×10^−11^) was composed of TFBs for the Zinc finger transcription factors EGRF (Early growth response family), E2FF (E2F-myc activator/cell cycle regulator) and ZF5F (binding site for the transcription factor Zfp161); (**Figure 1A** **)**. In addition, an alternative regulatory module of two elements (EGRF and E2FF) was found present in the promoter of six out of the seven TFs consistently up-regulated in the preeclamptic placenta (*P*≤8.76×10^−8^). In the case of the consistently down-regulated TFs we found one highly significant model (*P*≤1.99×10^−10^) composed of six elements corresponding to TFBS for E2FF, RXRF (Retinoid × receptor heterodimer-binding sites), KLFS (Kruppel-like factors) and ZF02 (C2H2 zinc finger transcription factors 2). This module was present in the promoter of three out of four genes (**Figure 1B**).

### Transcription Factors Interactome

**Figure 2:**
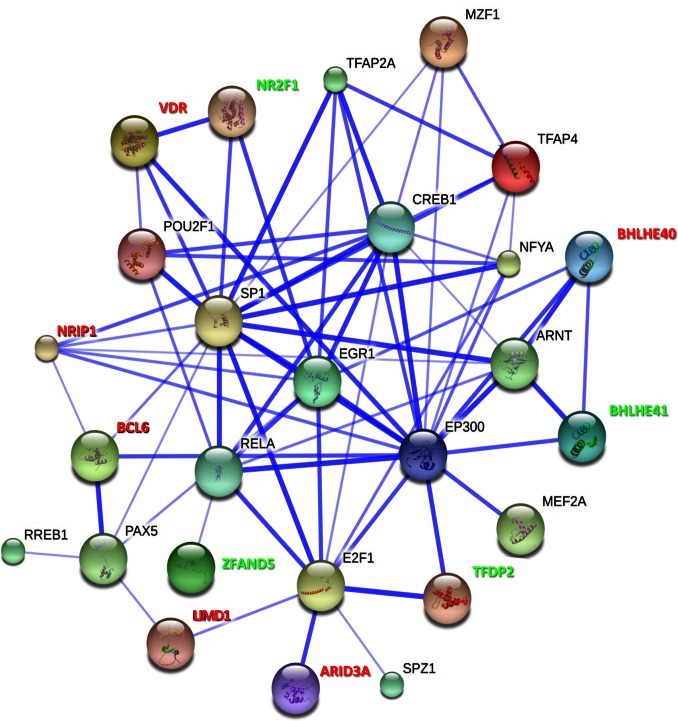
Transcription factors interaction network in the preeclamptic placenta. TFs modified in the preeclamptic placenta were analyzed with STRING v9.0 to identify putative interactions. Blue lines represent the evidence for the association. The thickness of the line is proportional to the confidence level of the interaction. TFS found to be modified at the transcriptional level in the preeclamptic placenta appear in red (up-regulated) and green (down-regulated).

We used the STRING database to search for known interactions among the TFs identified as consistently modified in the preeclamptic placenta and also with those identified through our TFBS analysis. Subsequently, we used the STRING functions to extend the network and display close interacting factors. As shown in **Figure 2** **,** the majority of the TFs modified in the preeclamptic placenta including those inferred from the TFBS analysis present a close functional association. In addition, we identified that the transcription factor EP300 (E1A binding protein p300) is connected with the largest number of preeclampsia-associated TFs in an extended interaction network.

## Discussion

The molecular basis of transcriptional alterations in the preeclamptic placenta remains elusive. Herein, we identified several TFs which are putatively involved in the regulation of genes that are consistently associated with PE. We started our analysis by intersecting publicly available datasets from microarrays analysis of preeclamptic placentas. This allowed building a consensus list of modified genes in the preeclamptic placenta. Of these, 67 were up-regulated and 31 down-regulated. The functional analysis identified several categories including: signaling, biological quality regulation, myeloid cell regulation, and cell proliferation among the up-regulated genes. Blood vessel regulation, blood circulation, cellular homeostasis and response to oxidative stress were the functional categories identified as enriched in the down-regulated genes. Consistently with preeclampsia pathophysiology, pathway analysis showed an over-representation of genes involved in peroxisome proliferative activated receptor alpha, lipid biosynthesis, hypoxia, and VEGF response. Subsequently, we extended our analysis by searching common TFs possibly involved in gene regulation in preeclamptic women’s placentas. Several bioinformatics tools detected over-represented TFBSs in the promoters of the PE-associated genes. Inside up-regulated genes promoters we found an over-representation of TFBSs for NFKB, SP1, RREB1, ARNT, CREB1 and AP-2. Conversely, among the down-regulated genes we found a prevalence of TFBSs for MZF-1, NFYA, E2F1 and MEF2A. Interestingly several transcriptionnally modified genes were themselves transcription factors. Below, we discuss the molecular mechanisms of action of all these TFs, and how they might be related to the placental dysfunction in the context of PE.

### 

#### NFkB

Belongs to the REL family of TFs which in mammals is composed of five members: RelA/p65, RelB, c-Rel, p50(NFkB1) and p52(NFkB2). NFkB proteins bind to kB sites as dimers, either homodimers or heterodimers, and can exert both positive and negative effects on gene transcription. Signaling mediated by NFkB stimulates inflammation, invasion, angiogenesis, and cell proliferation and it is also associated with apoptosis regulation [23]. NFkB is known to be involved in PE at several levels and in different cell types. Placental NFkB has been found activated nearly 10-fold in PE [24]. *In vitro* experiments show that oxidative stress, a hallmark of preeclamptic placenta, causes NFkB activation in a trophoblast-like cell line, which is enhanced by TNF-α [24]. In addition, trophoblast cells respond to TLR3 activation by signaling through both NFkB and IRF pathways resulting in expression of inflammatory mediators and, in particular, the PE-related anti-angiogenic factor sFLT-1 [25]. In endothelial cells (ECs) preeclamptic plasma up-regulates NFkB activity by 2.5-fold compared with normal plasma [26]. This results in ECs activation. Several factors in the preeclamptic plasma induce endothelial NFkB activation, including cytokines, lipid peroxides, peroxinitirites, and shed membrane microparticles (mainly derived from apoptotic cytotrophoblasts, leukocytes and platelets), [26], [27]. Increased endogenous activation of NFkB associated with TNF-α and IL-1β release has been detected in PBMC in PE as compared to normal pregnancies [28]. Several factors associated with PE have been shown to be able to induce NFkB activation including adiponectin, leptin, cytokines (TNF-α, IL-6), lipid peroxides, and agonistic auto-antibodies to the angiotensin II receptor type I (AT1-AA); [29], [30]. Moreover experiments studying placental ischemia-reperfusion *in vitro* and *in vivo* provide strong evidence indicating that oxidative stress and ROS production can activate the NFkB signalling pathway [31]. Activation of the NFkB pathway in the placenta, together with other stress signaling pathways (p38, MAPK, JNK), results in the placental production of inflammatory mediators, apoptotic debris, and anti-angiogenic mediators.

#### SP1

is a ubiquitously expressed Zinc Finger TF that regulates the expression of thousands of genes implicated in the control of cellular processes. SP1 is also involved in chromatin remodeling through interactions with chromatin-modifying factors such as EP300 and histone deacetylases (HDACs). Although constitutively expressed, phosphorylation, acetylation, sumoylation, ubiquitylation, and glycosylation influence the transcriptional activity and stability of SP1 [32]. In the placenta, SP1 is involved in the differentiation of the cytotrophoblast and regulates the expression of several proteins including ID-1, Syncytin, the AT1 receptor, the 11beta-hydroxysteroid dehydrogenase type 2 (11β-HSD2) and the pregnancy-specific glycoprotein 5, as well as several cullin genes involved in the dynamics of protein recycling [33]. Moreover, compound *Sp1/Sp3* heterozygous mice show severely reduced spongiotrophoblast layer and a disorganized labyrinth layer. Within the spongiotrophoblast layer both spongiotrophoblast cells and trophoblast glycogen cells are reduced. Haploinsufficiency of both Sp1 and Sp3 also leads to a severe disruption of the normal labyrinth layer architecture [34]. In response to oxidative-stress induced by hypoxia, SP1 becomes activated and induces the expression of several factors including VEGFA, β-enolase, cyclooxygenase 2, and carbonic anhydrase 9. SP1 is also involved in the inflammatory response and, together with NFkB and AP-1, up-regulates the expression of VCAM1 and ICAM1 adhesion molecules, tumor growth factor (TGF-β) and platelet-derived growth factor (PDGFβ), and, finally, monocytes chemotactic protein-1 (MCP1) and osteopontin cytokines (28).

#### AP2

The activator protein-2 (AP-2) family consists of five members, AP-2α, AP-2β, AP-2γ, AP-2δ, and AP-2ε, encoded by different genes. These isoforms can directly transactivate their target genes by binding the same GC-rich consensus sequence [35]. AP-2α and AP-2γ are expressed in the placenta, and they control syncytiotrophoblast-specific gene expression [36], [37]. In *AP-2*γ-deficient mice all derivatives of the throphoblast cells are formed, however both the embryo and the extraembryonic tissues are severely growth retarded. This growth retardation is based on a reduced proliferation of the cells of the ectoplacental cone and a reduced number of giant cells [38]. In addition, AP-*2*γ has been shown to regulate the genes for adenosine deaminase (*ADA*), human placental lactogen, and human chorionic gonadotropin-β [37], [39], [40]. The expressions of AP-2α and AP-2γ have been found elevated in the preeclamptic placentas in comparison with the gestational age-matched control placentas [41]. Moreover, the over expression of AP-2α or AP-2γ in an extravillous trophoblast (EVT) cell line, decreased its migratory and invasive abilities [41]. This was associated with reduced expression of protease activated receptor-1 and matrix metalloproteinases and a significant induction of plasminogen activator inhibitor-1 and the tissue inhibitor of metalloproteinase-1. The same study has shown that in this EVT cell line TNF-α (which is present at higher levels in PE) induces both AP-2α and AP-2γ expression. Thus, the over-representation of genes containing TFBS for AP-2 in our study is consistent with the reported increased expression of AP-2 in PE and its known role in trophoblasts genes regulation.

#### CREB1

The cAMP responsive element binding protein 1 (CREB1), a member of the leucine zipper family of DNA-binding proteins, is ubiquitously expressed and binds as a homodimer to the cAMP response element (CRE). In the placenta, CREB contributes to the regulation of PLGF gene expression [42]. Moreover in cytotrophoblast cells CREB, modulates human chorionic gonadotropin (hCG) gene-expression by a direct protein-protein interaction with AP-2α [43]. Also, a recent study has shown that hCG added to cytotrophoblast cells lines (JEG-3, BeWo) or to placental explants induces endogenous leptin expression. This induction appears to be mediated by CREB [44].

#### ARNT (HIF-1β)

ARNT (aryl hydrocarbon receptor nuclear translocator) is the beta subunit (HIF-1β) of the heterodimeric transcription factor, hypoxia-inducible factor 1 (HIF-1). HIF-1 is a ubiquitous TF complex involved in the regulation of the cellular responses to oxygen deprivation (hypoxia). Under normoxic conditions the HIF-1α subunit is constitutively transcribed, translated and hydroxylated at multiple proline residues. This hydroxylation targets HIF-1α for proteasomal degradation. In hypoxia, mitochondria-derived ROS inhibits HIF-1α hydroxylation, enabling nuclear translocation, heterodimerization with the constitutively expressed ARNT (HIF-1β), binding to DNA, interaction with the co-activators p300/CBP and subsequent activation of hypoxia–responsive genes. In the developing placenta ARNT (HIF-1β) plays a critical role in cell differentiation [45]. Moreover, as a component of the HIF-1 complex ARNT (HIF-1β) regulates the expression of placental genes responsive to hypoxia. Studies in both preeclamptic patients and animal models have revealed the existence of hypoxia in the preeclamptic placenta [46], [47], [48]. Hypoxia in PE, is believed to be the consequence of shallow invasion of the decidua by the cytotrophoblasts resulting in impaired remodeling of the spiral arteries. This leads to reduced uteroplacental blood flow causing placental hypoxia, oxidative stress, and inflammation. The analysis of placental explants and *in vitro* studies on cytotrophoblasts have shown that several factors involved in the maternal manifestations of the preeclamptic syndrome are transcriptionally regulated by the HIF-1 complex including: Endothelin 1 [49], [50], Endoglin [51], the antiangiogenic factor sFLT-1 [52], Leptin [53], and the vasoconstrictors Urotensin II [54], Urocortin-2 and Urocortin-3 [55]. Therefore, the fact that the analysis of the promoters of consistently modified genes in PE reveals and over-representation of HIF-ANRT binding sites is consistent with the central role played by hypoxia in the development of PE.

#### RREB1

is a zinc finger TF that binds to RAS-responsive elements (RREs) of gene promoters [56]. In the placenta, RREB1 is expressed in the extravillous cytotrophoblasts were it could be involved in pathological repression of the human leukocyte antigen G (HLAG). HLA-G is expressed in the human placenta and amnios, and plays an essential role in the maternal tolerance toward the fetus through the inhibition of the NK and T lymphocyte-mediated direct cytotoxicity. Both circulating HLA-G and HLA-G protein expressed in the extravillous cytototrophoblasts are reduced in PE [57], [58], possibly trough oxidative stress [59]. RREB1 can inhibit expression of HLA-G by binding to RREs within the HLA-G promoter [60]. RREB1 is also involved in the response to cellular stress as it binds to the p53 gene core promoter and up-regulates p53 transcription. One known effect of the oxidative stress in PE is to cause oxidative DNA damage [61]. Thus, it is tempting to speculate that RREB1 could activate p53 gene expression in the preeclamptic placenta. However, at present there are contradictory studies concerning the up-regulation of p53 in the preeclamptic placenta [62].

#### MZF1

Myeloid zinc finger 1 (MZF1) is a member of the SCAN domain family of TFs. MZF1 is preferentially expressed in hematopoietic cells, and may be involved in the transcriptional regulation of hematopoietic-specific genes [63]. A putative role in placental physiology or pathology is currently unknown. However, the human placenta has been recognized to work as a hematopoietic organ during the embryonic and fetal development [64]. Increased hematopoietic activity in the preeclamptic placenta has been suggested [65]. On the other hand MZF1, together with SP1 and ZBTB7B has been involved in the regulation of the SERPINA3 in the cytotrophoblastic cell line JEG3 [66]. SERPINA3 is a serine protease inhibitor known to be up-regulated in human placental diseases (including PE) in association with a hypomethylation of the 5' region of the gene [67]. Over expression of SERPINA3 in JEG-3 cells, decreased cell adhesion to the extracellular matrix and to neighboring cells, but protects them from apoptosis.

#### E2F1

The E2F family of TFs controls the expression of genes involved in cell proliferation, differentiation, apoptosis, and DNA repair. In the context of PE, a recent study has reported the up-regulation of E2F1, together with several genes involved in cell cycle progression, in peripheral blood mononuclear cells (PBMCs) isolated from severe preeclamptic women [68]. In our analysis we did not detect E2F1 among the consensus up-regulated genes in PE. However, we found that its partner, TFDP2, is down-regulated in the preeclamptic placentas. Thus down-regulation of TFDP2 might result in impaired DNA-binding of E2F1, and lead to the deregulation of genes controlled by the E2F1-TFDP2 complex. On the other hand, it has been reported that under hypoxic conditions E2F1 and p53 are up-regulated, and are able to down-regulate expression from the VEGF promoter [69]. The minimum VEGF promoter mediating transcriptional repression by E2F1, was found to be composed of an E2F1-binding site with four SP1 sites in close proximity. Of note, it is known that E2F1 and Sp1 proteins physically and functionally interact and show functional synergism in promoters having binding sites for both [70]. In ECs, E2F1 can induce the expression of FLT-1, KDR, and ANGPT2, through a mechanism involving VEGF stimulation, and both Histones and E2F1 acetylation [71]. Previous studies had shown that the expression of FLT-1 and KDR is regulated by Sp1 proteins. [72]. Thus, we find again the association between E2F1 and SP1 binding sites in the regulation of this antiangiogenic genes.

#### MEF2A

(Myocyte enhancer factor 2A) belongs to the MADS (MCM1, agamous, deficiens, SRF) family of TFs and plays a pivotal role in the development of various organ systems, including the cardiovascular system [73]. The implication of this TF in placental development or in preeclampsia has not been studied. However, its role in the control of gene expression in smooth muscle cells (SMCs) and ECs suggests that it might be involved in the vascularization of the placenta. In vascular SMCs, MEF2A has been shown to be activated via reactive oxygen species and p38 mitogen-activated protein kinase. This leads to the induction of the transcription factor KLF5 in response to angiotensin II [74]. KLF5 has been found consistently up-regulated in cardiovascular diseases [75]. Within ECs, shear stress stimulates induction of KLF2 *via* the MEK5/ERK5/MEF2 pathway, which ultimately leads to MEF2A binding to and transactivating the KLF2 promoter [76]. KLF2, has been reported to be essential for the anti-inflammatory and antithrombotic functions of the endothelium [77]. The mechanisms by which KLF2 achieve its anti-inflammatory function are multiple and include inhibition of NFkB, activator protein-1 (AP-1), and activating transcription factor 2 (AP-2). Thus, the ROS produced in preeclamptic placenta could be involved in the activation of MEF2A in SMCs. On the other hand in the ECs, MEF2A activation could be part of an adaptive response seeking to protect the cells against inflammation and thrombosis (two characteristics of PE).

#### NFYA

associates with a dimer composed of NF-YB, and NF-YC subunits, forming a trimer that binds to DNA. The complex recognizes the pentanucleotide CCAAT, a motif present in the promoter regions of many genes [78]. The DNA interaction of the complex occurs through NFYA, suggesting a role as the regulatory subunit. ROS play also an important role in NFY regulation [79]. When oxidized, NFYB forms homodimers remaining localized in the cytoplasms, as a consequence the formation of the trimer and subsequent DNA binding is impaired. NF-Y is known to interact with several TFs to mediate the synergistic activation of specific classes of promoters. The most frequent TFs partners of NFY include: SREBP, SP1, KLFs, OCT-1 and E2F1. NFY seems to be also involved in the response to cell stress. Thus, NFY directly controls the expression of TFs genes such as P53 (DNA-damage), XBP1, CHOP/DDIT3 (ER stress), and HSF1 (Heat shock), [78]. The role of NFY in the regulation of genes involved in the response to cell stress could represent a link between this TF and PE. In this sense, NFYA and OCT-1 (another TF which appears over-represented in our analysis) synergistically regulate a P53-independent induction of GADD45 subsequently to DNA-damage [80]. The GADD45 stress sensor protein has been suggested to be the link between placental stress and the pathogenesis of PE through the induction of FLt-1. Thus in stressed placental explants GADD45a initiated a signaling cascade culminating in FLt-1 induction [81].

In addition to the TFs identified by our bioinformatic TFBS analysis, some of the genes consistently modified in the preeclamptic placenta encode TFs. Among the up-regulated genes we found: LIMD1, BHLHE40, VDR, CEBPA, BCL6, ARID3A and NRIP1. Among the down-regulated genes: TFDP2, ZFAND5, BHLHE41, and NR2F1.

LIMD1 inhibits E2F-mediated transcription, and suppresses the expression of the majority of genes with E2F1-responsive elements [82]. The up-regulation of this TF in the preeclamptic placenta seems coherent with the detection of an over-representation of TFBS for E2F1 among the down-regulated genes. On the other hand, LIMD1 has been recently involved in the regulation of the hypoxia response through a mechanism involving HIF1-α degradation [83]. LIMD1 up-regulation in the preeclamptic placenta might result from a feed-back mechanism aiming to regulate the transcriptional activity of the HIF complex. BHLHE40 (DEC1/STRA13) is another TF up-regulated in PE, known to be expressed in the cytotrophoblasts and fibroblast cells of the placenta [84]. Its gene expression is regulated by various extracellular stimuli, such as growth factors, serum starvation, hormones, nutrients, cytokines, and hypoxia through HIF-1α activation. CEBPA (CCAAT/enhancer-binding protein alpha) coordinates proliferation arrest and the differentiation of trophoblastic cells [85]. CEBPA is known to activate the expression of the leptin gene [86]. Thus, the up-regulation of CEBPA is probably related to the increased expression of leptin (one of the most consistently modified genes in the PE placenta). BCL6 mediates transcriptional repression and interacts with components of histone deacetylase co-repressor complexes including N-CoR and SMRT [87]. It is involved in a multiple biological processes including: regulation of inflammatory response; negative regulation of cell growth; negative regulation of transcription, response to DNA damage stimulus, negative regulation of B cell apoptosis. It has been speculated that up-regulation of BCL6 in the preeclamptic placenta could be related to deregulated DNA-damage response, cell cycle arrest, cell survival and immune response in trophoblast cells [5]. ARID3A is a nuclear matrix-associated TF that stimulates immunoglobulin heavy chain (IgH) expression and Cyclin E1/E2F-dependent cell cycle progression. [88], [89]. NRIP1 (also known as RIP140) has been shown to bind and repress the transcriptional activity of several nuclear receptors including the estrogen receptors, the peroxisome proliferator-activated receptors, the vitamin D receptor, thyroid hormone receptors, and estrogen-related receptors [90]. NRIP1 has a major role as co-regulator of genes involved in lipid and glucose metabolism, in heart, skeletal muscle, and liver. Its biological role in the placenta is currently unknown. However, we found in our study that the most significantly up-regulated pathways concern the peroxisome proliferators-activated receptor and lipids biosynthesis. Its implication in placental inflammation through its cooperation with NFkB is also possible. TFDP2 is a member of the E2F/DP family [91]. As mentioned above, it binds DNA cooperatively with E2F family members. The down-regulation of TFDP2 implies impaired E2F1 driven transcription, and seems to be coherent with the fact that TFBS for E2F1 are over-represented among the down-regulated genes in the PE placenta. ZFAND5 plays a role in the regulation of NFkB activation and apoptosis. Over-expression of ZFAND5 sensitizes cells to TNF-induced apoptosis [92]. BHLHE41 (DEC2/SHARP) is associated with the regulation of apoptosis, circadian rhythm and the response to hypoxia [93]. This TF binds to HIFs and promotes HIF proteasomal degradation by serving as the HIF-presenting factor to the proteasome independently from pVHL (von Hippel-Lindau tumor suppressor), hypoxia and the ubiquitination machinery. BHLHE41 therefore determines the intrinsic instability of HIF proteins to act in parallel to, and cooperate with, oxygen levels [94]. Therefore down-regulation of BHLHE41, is probably related to the up-regulation of hypoxia responsive genes in the PE placenta. NR2F1 (COUP-TFI) is a member of the orphan subfamily of nuclear receptors required for multiple physiologic and biologic functions, including heart and vascular system function and cholesterol/lipid homeostasis [95]. Little is known about a putative role of NR2F1 in the placenta. A study identified NR2F1 as a repressor of the hLHR (Luteinizing hormone receptor) gene transcription in JAR cells (issued from a human placental choriocarcinoma), [96]. In the placenta LH mediates gonadotropin signals and triggers intracellular responses that participate in maturation and function of the gonads as well as the regulation of steroidogenesis and gametogenesis. Nevertheless, we observe that TFBS for COUP are over-represented in the list of down-regulated genes in the PE placenta.

Another TF worth mentioning here is STOX1 (storkhead box 1). To date only two PE susceptibility genes have been identified (ACVR2A and STOX1). Of these, STOX1 encodes a winged-helix TF showing great similarity with the FOX family of TFs [97]. STOX1 has been found to be involved in trophoblast dysfunction in PE. Over-expression of STOX1 in the JEG-3 choriocarcinoma cell line (as a model for trophoblasts), deregulates many genes which are also modified in the preeclamptic placenta [98]. Transgenic mice over-expressing the human version of STOX1 develop a syndrome similar to severe human PE. During pregnancy, the mice undergo a steep increase in blood pressure, develop proteinuria and renal histology reveals accumulation of fibrin [99]. Here, we have compared the transcriptome of the JEG-3 cells over-expressing STOX1 and the list of consistently modified genes in PE and found a significant correlation (data not shown). Genes such as LEP, ENG, EBI3, FSTL3, SPAG4, LHB, TMEM45A, GCLM, TFDP2, or TSPAN12 that we find consistently modified in PE, are also transcriptionally modified in the JEG-3 over-expressing STOX1. The microarrays analyzed in the present study do not reveal any significant modifications in the transcriptional levels of STOX1. However, STOX1 is known to be post-transcriptionally regulated. When phosphorylated by Akt, the STOX1 protein is inhibited from entering the nucleus and subsequently degraded by ubiquitination. In the absence of phosphorylation STOX1 is addressed to the nucleus [97]. As the STOX1 DNA-binding domain shows great similarity to FOX transcription factors it has been proposed that STOX1 binds to the FOX binding sites in the promoters of target genes. In our analysis FOX binding sites are detected as over-represented among the consistently down-regulated genes.

Having identified a set of TFs which are likely involved in the transcriptional modifications of the preeclamptic placenta, we investigated the putative interactions within them. The interactomics analysis using the STRING software showed that most of these TFs present close interactions. Moreover, by extending the interaction network we found that many of them were strongly connected with a pivotal TF: EP300. This protein is ubiquitously expressed and functions as a scaffolding actor between the TFs and the RNA polymerase II. It functions also as a histone acetyltransferase that regulates transcription via chromatin remodeling [100]. Among others, it mediates cAMP-gene regulation by binding specifically to phosphorylated CREB protein [101]. EP300 has been also identified as a co-activator of HIF-1α, and, thus, plays a role in the stimulation of hypoxia-induced genes such as VEGF [102]. The loss of one functional copy of the gene causes a rare disease in infants, the Rubinstein-Taybi syndrome. This disease is characterized by growth retardation, dysmorphic features, skeletal abnormalities and mental retardation [103]. Interestingly, three of the babies out of the seven reported cases, were born from women who developed preeclampsia during the pregnancy [104]. This suggests that there could be an association between EP300 heterozygotic deleterious mutations and PE. The interaction of EP300 with most of the TFs identified in our study enhances its possible implication in PE.

In summary, our study has identified a number of TFs which could be key regulators of the changes in gene expression observed in the preeclamptic placenta. This allows developing hypothesis about the molecular mechanisms at work in the diseased placenta. However, there are a number of limitations of our study which must be taken into consideration. We have drawn a list of consistently modified genes in PE from the publicly available microarray data sets. That corresponds to only six studies from a total of 20 published microarray studies on preeclampsia. Unfortunately, the datasets corresponding to the majority of studies have not been deposited in public databases. Moreover, the authors do not provide in their manuscripts complete lists of modified genes. The access to more datasets would have increased the statistical power of the study, and presumably identified even more striking commonalities. Another aspect to consider is that these microarray experiments were done on placental samples which are composed of different cell types. This heterogeneity can cause noise that disturbs the correct prediction of a co-regulated gene set, and hence of the TFs involved in their regulation. Finally, we arbitrarily chose to limit the size of the promoters to be analyzed to 1200 bp. We postulated that the TFs regulating the activity of the modified genes would bind TFBS close to the TSS (-1000/+200 bases). If we had chosen other promoter lengths we might get different results. In a previous study published in 2006, Vasarhelyi et al analyzed the promoters of genes found to be modified in preeclamptic placentas [105]. They reported an over-representation of TFBS corresponding to NFkB(p50), SREBP and E47. Except for NFkB, the TFs identified in their study are different to those reported here, these differences being probably due to the data used for the studies. Vasarhelyi et al extracted data from a number of studies performed between years 2002 to 2005 [47], [106], [107], [108]. At that time, microarrays offered only a partial covering of the human genome. Thus, we used more recent data corresponding to microarrays with full coverage of the human genome.

Despite all this caveats our study allowed to identify a number of TFs involved in PE. Although a few of them are found to be consistently modified in the preeclamptic placenta at the transcriptional level, many of the TFs identified by our study (NFkB, CREB, ARNT, SP1, E2F1, NFYA…) are regulated by post-transcriptional mechanisms. These post-transcriptional modifications (acetylation, methylation, phosphorylation, sumoylation, etc… ), can be triggered by cellular stresses which are known to be associated with PE such as hypoxia, inflammation, oxidative stress, DNA-damage, etc… The validity of the hypothesis raised by our bioinformatic study need to be confirmed by experimental studies analyzing the implication of these TFs (including their post-transcriptional modifications) in both, *in vitro* models and *in vivo* in preeclamptic placentas.

## Supporting Information

Table S1 Complete list of consistently up-regulated genes in the preeclamptic placenta. The lists of up-regulated genes for each of the six preeclamptic placenta microarrays analyzed in this study were confronted using the GENOMATIX list comparison tool (Genomatix Software GmbH, Munich, Germany). This allowed to identify those genes which were consistently up-regulated. Those showing similar modification in at least 4 studies were considered relevant and included in a final list of consistently up-regulated genes.
(XLSX)

Table S2 Complete list of consistently down-regulated genes in the preeclamptic placenta. The lists of down-regulated genes for each of the six preeclamptic placenta microarrays analyzed in this study were confronted using the GENOMATIX list comparison tool (Genomatix Software GmbH, Munich, Germany). This allowed to identify those genes which were consistently down-regulated. Those showing similar modification in at least 4 studies were considered relevant and included in a final list of consistently down-regulated genes.
(XLSX)
